# A glance at the non-technical skills of nurses: simulation contributions

**DOI:** 10.1590/1518-8345.0000.2791

**Published:** 2016-12-08

**Authors:** Emilia Campos de Carvalho

**Affiliations:** Full Senior Professor, Escola de Enfermagem de Ribeirão Preto, Universidade de São Paulo, PAHO/WHO Collaborating Centre for Nursing Research Development, Ribeirão Preto, SP, Brazil. E-mail: ecdcava@usp.br

**Figure:**
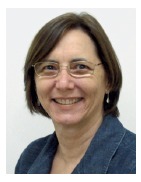

The Nursing area is increasingly faced with the challenge to prepare its professionals for the performance of technical and non-technical skills. In this sense, simulations, particularly high-fidelity ones, have been recognized as allies, with advantages for patient safety, teamwork, cost reduction in the real scenario and the handling of emotions of the learners. 

Herein, we are interested in considering the non-technical skills, whose relevance is based in knowing that a significant portion of the adverse events^1^ of these skills are attributed to non-compliance with quality.

 The term non-technical skill, which comes from the aviation area in the '90s and is used in different areas, including health, refers to the cognitive and social skills and the personal resources that complement the technical skills and contribute to the safety and effective performance of tasks. Or, in the words of authors of the area of health^1^ ^-^ ^2^, it covers the preparation or knowledge of the situation, decision-making or problem-solving, leadership, teamwork, communication and management of stress and fatigue.

Successful teaching examples reinforce the use of simulations, in various scenarios and in varying degrees of complexity, as in nurse-patient communication development, interprofessional relations in critical situations or emergencies, teamwork, communication of bad news, ethical dilemmas, intra-team conflicts, management of stressful situations, leadership exercises, among others. Therefore, they point to development opportunities in the attitudinal, behavioral, ethical and moral fields. 

In addition to the clinical scenarios traditionally used in simulations for the development of non-technical skills, other forms of simulation, involving games or virtual environments, are mentioned in the literature. The contribution of Second Life^3^ is highlighted, an open virtual environment of free access that is still little used in Nursing.

One of the crucial points in the teaching or training of skills, especially non-technical ones, is their assessment. Models for this end have pointed to the use of indicators of (observable) behaviors. A recent review of the literature^4^ has identified several methods to measure observable behavior, but the authors considered that such methods were not validated or their properties were not strongly supported, what led them to suggest the need for the development of reliable systems for the training of professionals.

Some of the currently known assessment tools in the area of health follow the recommendations of the European taxonomy of pilots' non-technical skills (NOTECHS)^5^, having the categories of cooperation, leadership and managerial skills, perception of the situation and decision making, each subdivided into behavioral markers and elements.

We highlight that both the instruments created for the medical area, which assess professional behaviors in anesthesia and surgery procedures or the perceptions of interactions between the team during surgery, among others, and those instruments created by nursing, which assess the clinical judgments of nurses, among others, are relevant and contribute for the assessment of non-technical skills in simulation situations, especially if accurate and composed of observable indicators.

However, there is still a long way to go regarding the training and assessment of these skills in the context of large-scale events, such as accidents or natural disasters, in particular for the development of behaviors or skills of collaboration, negotiation and communication^6^.

The familiarization with such a teaching technique and the tools to assess these skills, essential to the professional exercise, taking advantage of their potential, has been a challenge for the area of health, in particular nursing. It is up to trainers and institutions to both acknowledge such contributions and prepare themselves for their effective use.
